# Comparative cytogenetic analysis of marine *Palaemon* species reveals a X_1_X_1_X_2_X_2_/X_1_X_2_Y sex chromosome system in *Palaemon elegans*

**DOI:** 10.1186/s12983-017-0233-x

**Published:** 2017-10-12

**Authors:** Zeltia Torrecilla, Andrés Martínez-Lage, Alejandra Perina, Enrique González-Ortegón, Ana M. González-Tizón

**Affiliations:** 1 Grupo de Investigación en Biología Evolutiva (GIBE), Centro de Investigaciones Científicas Avanzadas (CICA), Departamento de Biología, Facultad de Ciencias, Universidade da Coruña, Campus A Zapateira, 15071 A Coruña, Spain; 2 Instituto de Ciencias Marinas de Andalucía (ICMAN, CSIC), Campus Universitario Río San Pedro, 11510 Puerto Real, Cádiz, Spain; 3 International Campus of Excellence of the Sea (CEI-MAR), Edificio Hospital Real, 11003 Cádiz, Spain

**Keywords:** Comparative cytogenetics, Decapoda, FISH, Karyotype, *Palaemon elegans*, *Palaemon serratus*, Sex chromosomes

## Abstract

**Background:**

The maintenance of species and the promotion of speciation are closely related to chromosomal rearrangements throughout evolution. Decapoda represents the most species-rich order among crustaceans and, despite its ecological and economic importance, little is known about decapod karyology. We aim at cytogenetically characterizing two sympatric prawn species.

**Results:**

Analysis of mitotic metaphases and meiotic diakinesis of the common prawn *Palaemon serratus* and the rockpool prawn *P. elegans*, revealed considerable differences between their karyotypes including chromosome numbers and sex determination systems. The cytogenetic data for *P. serratus* showed a diploid number of 56 and the putative absence of heteromorphic sex chromosomes. However, the diploid chromosome number in *P. elegans* was 90 for females and 89 for males. The karyotype of the females consisted of the three largest acrocentric pairs and 42 submetacentric and metacentric pairs, while the karyotype of the males comprised a clearly identifiable large metacentric chromosome and two acrocentric pairs as well as the smaller 42 pairs. These results highlight the presence of the X_1_X_1_X_2_X_2_/X_1_X_2_Y multiple sex chromosome system in *P. elegans*, which constitute the only sexual system for Decapoda reported cytogenetically using modern techniques. The origin of this sex chromosome system is discussed. We hypothesize that the chromosome evolution within the genus could involve several fusion events giving rise to a reduction on the chromosome number in *P. serratus*. In both species, the major ribosomal genes were located in two chromosome pairs and hybridization signals of the telomeric sequences (TTAGGG)_n_ were visualized at the telomeres of all chromosomes. C-banding revealed that, when present, constitutive heterochromatin had a predominantly telomeric distribution and no centromeric constitutive heterochromatin was observed.

**Conclusions:**

Although more comparative cytogenetic analyses are needed to clarify our hypotheses, the findings of this work indicate that the prawns of the genus *Palaemon* represent a promising model among Decapoda representatives to investigate the karyotype evolution and the patterns of sex chromosome differentiation.

## Background

Decapoda is the most species-rich order within Crustacea. This extremely diverse group plays a key role in the aquatic trophic relationships [1, 2] and many of these species have a significant commercial importance since they are exploited for human consumption in different countries around the world [3, 4]. However, despite the importance of this group, the limited knowledge of decapod crustacean karyology constitutes an obstacle to elucidate different modes of sex determination, the occurrence of chromosomal rearrangements along their evolution or clarify phylogenetic relationships between related species. To our knowledge, during the last 25 years karyological data have only been reported in 46 species of decapods belonging to 10 families (for a review, see [5]). This scarcity of studies is mostly caused by decapod chromosomes peculiarities, usually small-size, numerous and highly condensed [6].

The family Palaemonidae comprises 981 species [7] of which only 13 belonging to three genera (*Palaemon*, *Exopalaemon* and *Macrobrachium*) have been studied at the cytogenetic level. These species show a wide karyotypic diversity and remarkable differences in their diploid chromosome number (Table 1). The existence of sex chromosomes was never determined cytogenetically in any species of the genera of Palaemonidae family and only rarely in Decapoda.

**Table 1:** Chromosome numbers in the members of the family Palaemonidae

Species	Chromosome number	Reference
*Palaemon serratus*	2n = 56	[5]
*Palaemon khori*	2n = 96	[36]
*Palaemon elegans*	2n = 89♂/90♀	This study
*Exopalaemon modestus*	2n = 90	[34]
*Exopalaemon carinicauda*	2n = 90	[35]
*Macrobrachium carcinus*	2n = 94	[59]
*Macrobrachium superbum*	2n = 100	[60]
*Macrobrachium siwalikensis*	2n = 100	[61]
*Macrobrachium nipponense*	2n = 104	[62]
*Macrobrachium idella*	2n = 104	[63]
*Macrobrachium scabriculum*	2n = 104	[64]
*Macrobrachium lamarrei*	2n = 118	[65]
*Macrobrachium rosenbergii*	2n = 118	[65]
*Macrobrachium villosimanus*	2n = 124	[66]

The genus *Palaemon* Weber, 1795 (Crustacea: Decapoda) is a group of caridean prawns of the family Palaemonidae. Recently, phylogenetic and taxonomic revisions changed the status of the genus *Palaemon* [8–11] as well as the number of its species. The genus *Palaemon* currently comprises 86 species, two of which have been recently described (*Palaemon minos sp. nov*. and *Palaemon colossus sp. nov.*) [10].

The selected species, the common prawn *P. serratus* and the rockpool prawn *P. elegans*, have a wide geographical distribution from the North Sea to Mauritania and Namibia, respectively, including the Mediterranean and Black Seas [12, 13]. These species differ in physiology, life history strategies and larval development [14–16]. They are both marine prawns, but whereas *P. serratus* inhabits estuaries in the reproductive season, *P. elegans* is common in tidal rockpools, *Zostera*, *Posidonia* and *Cymodocea* meadows and it also can be found in slightly brackish water close to river mouths [17].

Whilst the species are morphologically similar, it is unknown whether they share chromosome number and morphology. The karyotype of *P. serratus* was recently described. In our previous study, the karyotype of *P. serratus* was described [5].

Here, we aim at: (i) extending the previous knowledge on the cytogenetics of *P. serratus*; (ii) providing the first karyological data for *P. elegans* and compare them with what is known about *P. serratus* and (iii) identifying their sex chromosome systems. For this purpose we have studied the mitotic and meiotic chromosomes of both species and applied conventional staining and banding techniques, fluorescence in situ hybridization (FISH) with 18S–5.8S-28S rDNA and telomeric (TTAGGG)_n_, (TTAGG)_n_ and (TAACC)_n_ probes.

## Methods

### Biological material and chromosome preparation

Specimens of *P. serratus* and *P. elegans* used in this study were collected from the Artabro Gulf (43° 25′N, 8°20′W) in the northwest of Spain. Animals were captured with a fish trap and carried alive to the laboratory. Animals were kept at 18 °C in an aerated aquarium and fed with frozen brine shrimp for 24 h. Individuals were sorted into species [13] and the sex was determined by the presence (in males) or absence (in females) of the masculine appendix on the endopodite of the second pleopod [18]. Metaphase chromosome spreads were obtained according to previously described protocol [5]. Briefly, adult shrimps were injected at the epimeral line with 0.005% colchicine solution (5 μl/g body weight) 3–5 h before anesthetization by exposure to ethyl ether. Cefalothorax content (including gonad, circulatory tissue, digestive tissue and muscular tissue) was removed from each individual and then immersed into a hypotonic solution of 0.56% KCl for 10 min at room temperature. The tissue was then fixed four times in freshly prepared ethanol/glacial acetic acid (3:1) for 20 min each time at 4 °C, followed by overnight incubation in a fresh fixative at 4 °C. The following day a piece of about 3 mm of the heterogeneous fixed material was dissolved in 45% acetic acid and a cell suspension was obtained. Then, 4–5 drops of this suspension were pipetted onto pre-heated slides at 43 °C and air-dried.

### Chromosome staining and fluorescence in situ hybridization

The slides were stained with anti-fade medium Vectashield (Vector Laboratories) containing 1.5 μL/mL 4′, 6-diamidino-2-phenylindole (DAPI). C-banding was performed on metaphase plates following Sumner [19].

To locate the position and number of the 18S–5.8S-28S rDNA sites we used the DNA probe pDm 238 from *Drosophila melanogaster* [20] labeled with FITC by using Prime-It Fluor fluorescence labeling kit (Stratagene) following the manufacturer’s instructions.

Chromosome mapping of the telomeric sequences was carried out using a (TTAGGG)n Cy3-labeled pan-telomeric probe (Cambio) according to the instructions of the manufacturer; a PCR generated pentanucleotide (TTAGG)n repeat according to Ijdo et al. [21] labeled with rhodamine-dUTP and the (TAACC)_2_ probe was synthesized and directly 5′ labeled with Cy3 (Isogen Life Science).

In situ hybridization was performed as described in González-Tizón et al. [22] with minor pre-hybridization and post-hybridization modifications. The slides were pretreated with DNAse-free RNAse (100 μg/mL in 2 x SSC) for 30 min at 37 °C, washed in 2 x SSC for 5 min and dehydrated in a graded ethanol series. Post-hybridization washes consisted of two 5-min incubations in 2 × SSC at 37 °C and at room temperature, respectively, followed by a 5-min incubation wash in 0.1 M Tris, 0.15 M NaCl and 0.05% Tween-20 at room temperature. Chromosomes were counterstained with 40 μL of anti-fade medium Vectashield containing 1.5 μL/mL DAPI.

Images were captured using a Nikon Microphot-FXA epifluorescence microscope equipped with a Nikon DS-Qi1Mc digital camera and processed with the NIS-Elements D 3.10 software.

The cytogenetic analyses described above were performed on *P. serratus* and *P. elegans* with the exception of the 45S rDNA chromosomal location in *P. serratus*, characterized in a previous work [5].

## Results

### Karyotypes, heterochromatin distribution and Fluorochrome staining

Mitotic and meiotic metaphases were obtained from 18 *P. elegans* specimens (8 females and 10 males) and 10 *P. serratus* specimens (6 females and 4 males). At least 15 metaphases per individual were observed, specifically 126 in *P. elegans* females, 153 in *P. elegans* males, 92 in *P. serratus* females and 31 in *P. serratus* males.

**Fig. 1:**
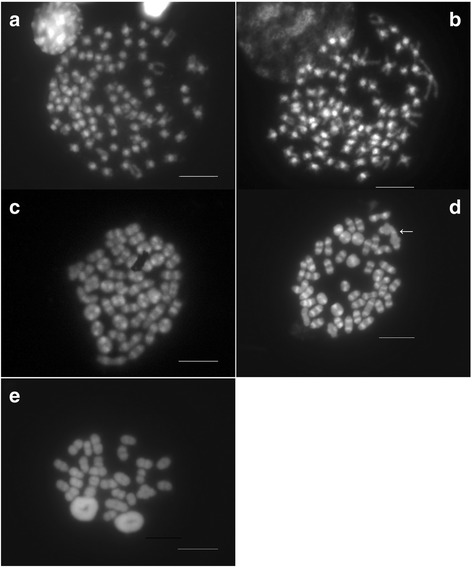
Metaphase plates of *P. elegans* (**a**) female and (**b**) male. Meiotic diakinesis of *P. elegans* (**c**) female and (**d**) male; the arrow shows the sex trivalent. (**e**) Meiotic diakinesis of *P. serratus* male. The bar equals 10 µm

**Fig. 2:**
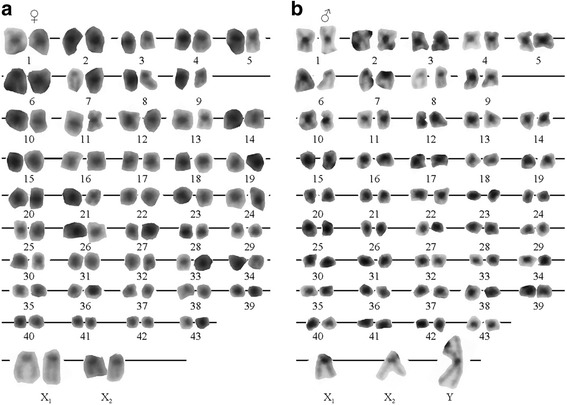
*Palaemon elegans* karyotypes. Female (**a**) and male (**b**)

The diploid chromosome number in *P. elegans* was 90 for females and 89 for males (Fig. 1a, b; Table 1). The karyotype consisted of 43 autosomal chromosome pairs: 5 metacentric/submetacentric, 4 subtelocentric/telocentric, and 34 hardly distinguishable due to size similarities (Fig. 2). The karyotype of the females also included two large telocentric sex chromosome pairs (Fig. 2a), while that of the males included one clearly identifiable large metacentric chromosome and two telocentric chromosomes (Fig. 2b). Thus, male heterogamety is evidenced by a metacentric chromosome present only in the male karyotype (Y chromosome) which is the largest element of the complement. During meiotic diakinesis, each arm of the large metacentric Y is terminally associated with one acrocentric chromosome (X_1_ and X_2_) forming a trivalent (X_1_X_2_Y, Fig. 1d). Therefore, in diakinetic plates males exhibited 43 autosomal bivalents and one sex trivalent while females showed 45 undistinguished bivalents (Fig.1d, c).

In *P. serratus*, the karyotype was identical to that previously described (2n = 56) [5]. At meiotic diakinesis 28 bivalents in both sexes were observed (Fig. 1e).

Fluorochrome staining with DAPI revealed bright centromeric/pericentromeric AT-rich blocks on all chromosomes in *P. elegans* and *P. serratus* (Fig. 1) whereas interstitial bands were observed on the four largest chromosomes of *P. serratus*. In *P. elegans* chromosomes DAPI-bands were noticed in some terminal regions, always weaker than those found at the centromeres. We also detected large telomeric DAPI faint segments in a few chromosomes.

**Fig. 3:**
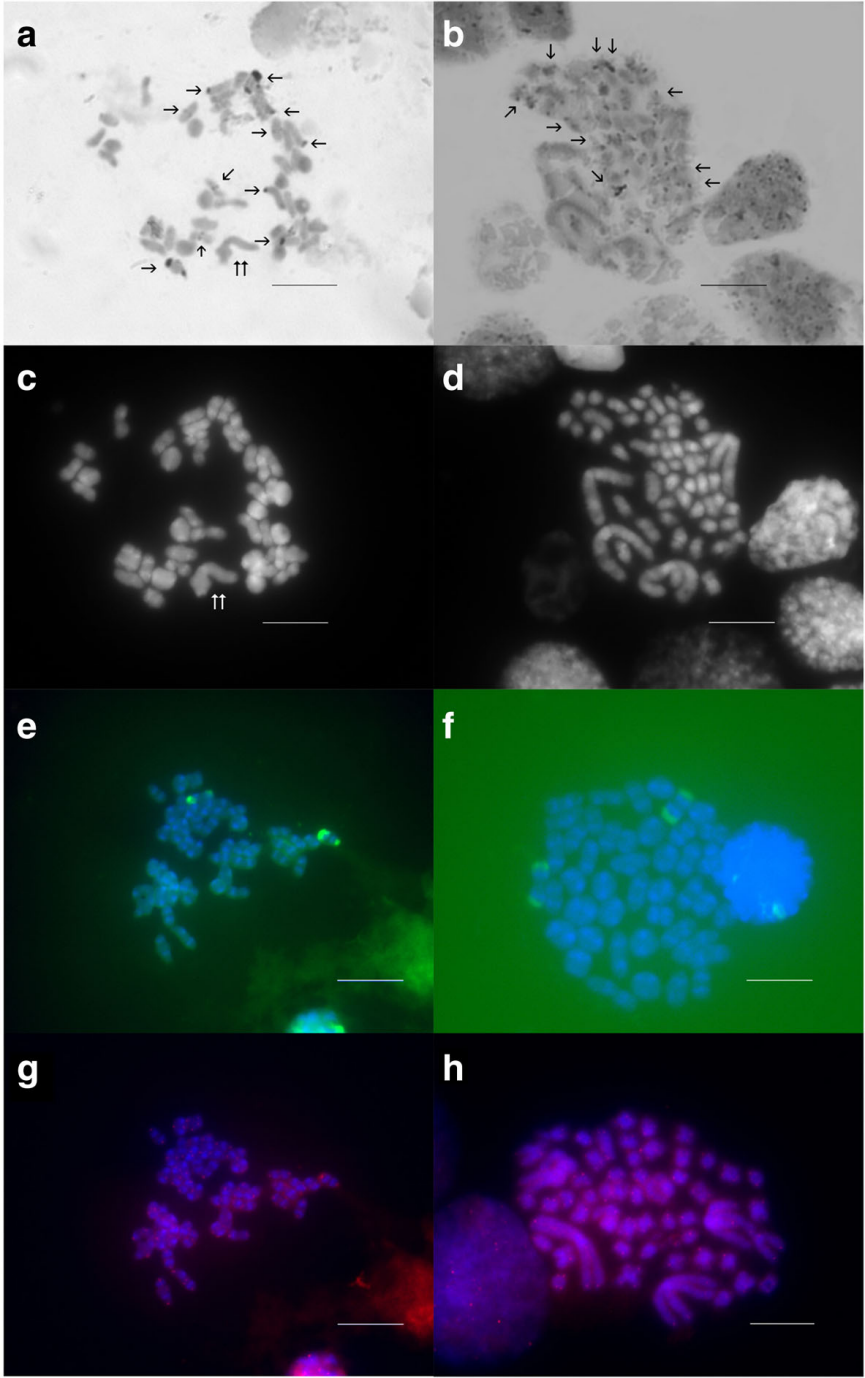
C-banded plates of (**a**) meiotic diakinesis of *P. elegans* and (**b**) mitotic metaphase of *P. serratus* males. Single arrows show C-band blocks, double arrow shows the sex trivalent (**c**, **d**) The same meiotic diakinesis of *P. elegans* and mitotic metaphase of *P. serratus* males, stained with DAPI. Chromosomal localization of the 18S–5.8S-28S rDNA genes of (**e**) *P. elegans* male and (**f**) female. Chromosomal localization of the (TTAGG)n telomeric sequences in (**g**) *P. elegans* male and (**h**) *P. serratus* male. Asterisks in *a* and *c* indicate the sex trivalent. The bar equals 10 μm

C-banding revealed that, when present, constitutive heterochromatin had a predominantly telomeric distribution in both species of *Palaemon* (Fig. 3a, b). Furthermore, no centromeric constitutive heterochromatin was observed. A large heterochromatic block was also found in the telomeres of four small size chromosomes in both species. In *P. elegans* the two X chromosomes and the Y chromosome were C-negative (Fig. 3a). In *P. serratus*, small weak bands of heterochromatin were also localized in interstitial positions of the large metacentric chromosomes. Slides containing C-banded chromosomes were previously stained with DAPI (Fig. 3c, d).

### Chromosomal mapping of the 18S–5.8S-28S rDNA genes

In situ hybridization of the 18S–5.8S-28S rDNA genes on meiotic chromosomes of both sexes of *P. elegans* revealed four sites of probe hybridization (Fig. 3e, f). The rDNA probe mapped the free telomeres of two bivalents paired at one end (dumbbell-shape bivalents). Both 18S–5.8S-28S rDNA-bearing chromosome pairs were heteromorphic showing different hybridization intensity of the homologous chromosomes. FISH signals coincided with the heterochromatic blocks observed.

### Chromosomal location of the telomeric probes

In situ hybridization of the (TTAGGG)_n_, (TTAGG)_n_ and the (TAACC)_n_ telomeric sequences were made in *P. serratus* and *P. elegans*. No hybridization signals were detected with the (TTAGGG)_n_ or the (TAACC)_n_ probes while FISH with the (TTAGG)_n_ pentanucleotide repeat produced discrete fluorescence signals at the telomeres of all chromosomes in *P. serratus* and in all the diakinetic bivalents in *P. elegans* (Fig. 3g, h).

## Discussion

### Chromosome number and karyotypes

The diploid chromosome number obtained in this study for *P. elegans* falls within the range of the published chromosome numbers in other members of the family Palaemonidae, with *P. serratus* displaying the lowest number in the family (2n = 56).

The lack of cytogenetic studies in other members of the genus *Palaemon* hinders the definition of clear trends in karyotype evolution in these species. However, some evidence supports the hypothesis that the chromosome evolution within the genus could involve several fusion events giving rise to a reduction on the chromosome number in *P. serratus*: i) We observed interstitial DAPI-bright bands on the large metacentric chromosomes of *P. serratus*, being DAPI-positive bands that are characteristic of centromeric regions in both *Palaemon* species, as observed in other families of decapods such as Astacidae [23, 24], Cambaridae [25], Nephropidae [26], Scyllaridae [27] and Palinuridae [28]. ii) The presence of interstitial C-bands on these chromosomes may represent a chromosome fusion event. In general, decapod species on which this technique has been performed to date showed positive C-bands at the centromeres of almost all chromosomes (e.g. [29–32]), with the only exception of *P. serratus* and *P. elegans* wherein heterochromatin is located, mainly, in the telomeres. Macgregor and Sessions [29] postulated that the heterochromatin expansion is originated in the centromeres and then is dispersed towards the telomeres. Hence, according to this theory, dispersed distributions of heterochromatin (interstitial or telomeric) have an older phylogenetic status. Iii) Recent molecular phylogenetic studies have suggested that genus *Exopalaemon* should be included within *Palaemon* [8, 33]. Among *Exopalaemon*, karyological analysis of *E. modestus* and *E. carinicauda*, have shown a diploid chromosome number of 90 [34, 35]. More recently, the determination of the *Palaemon khori* karyotype was performed showing 2n = 96 [36].

In the light of our results, the phylogeny and the chromosome numbers found in the family Palaemonidae (Table 1), it seems likely that the high chromosome number detected represents the ancestral condition in this lineage whereas the reduced chromosome number of 2n = 56 observed in *P. serratus* constitutes a derived character. According to that, it seems plausible that the fusions constitute the main mechanism responsible for the origin of the *P. serratus* karyotype, which was also suggested for Astacidae and Parastacidae among Decapoda [24]. Further cytogenetic studies are still necessary in order to determine the mechanisms underlying the karyotype evolution in this group of species.

### Ribosomal loci

As previously reported in *P. serratus* [5], *P. elegans* revealed four sites of 18S–5.8S-28S rDNA probe hybridization corresponding to two loci. Given the divergence observed between both karyotypes, this may constitute a plesiomorphic condition for genus *Palaemon*. In all cases, the ribosomal clusters were located in terminal positions on two small chromosome pairs. In addition, conspicuous heterochromatin blocks were located in the major ribosomal genes sites, closely related to large telomeric DAPI faint segments, highlighting the rDNA GC-richness as reported for a wide variety of organisms (e.g. [37] and references therein).

Moreover, in *P. elegans* both rDNA-bearing chromosome pairs showed heteromorphism in size of the 18S–5.8S-28S rDNA locus between homologous as observed in males of some species of the Astacidae [23, 24]. Mlinarec et al. [24] have speculated from these findings that the heteromorphic chromosome pair could represent male sex chromosomes suggesting the presence of an XX-XY sex determination system, even though the karyological characterization of females is a pending issue. Conversely, our results show that in *P. elegans* the heteromorphic rDNA-bearing chromosome pairs correspond to autosomes, which have been reported for many animal groups (e.g. [38–41]).

### Telomeric repeats

This study shows for the first time the presence of the TTAGG repeat, known as the ancestral motif of arthropod telomeres, in the family Palaemonidae [42]. Since the presence of this repeat has not been demonstrated in most decapod families, it is interesting to confirm the constant presence of this motif within Decapoda, particularly when some animal groups have lost the TTAGG repeat during their evolution such as the crustacean species *Asellus aquaticus* (Isopoda) [43].

FISH with the (TTAGGG)n probe found in all vertebrates [44] and the (TAACC)n probe identified in the shrimp *Penaeus vannamei* [45] gave no hybridization signals. On the contrary, in both *P. serratus* and *P. elegans*, the hybridization signals of the (TTAGG)n probe were located at the telomeres of all chromosomes. Nonetheless, no interstitial telomeric signals were found as evidence of structural reorganizations occurring throughout chromosomal evolution. However, the fusion sites of ancestral chromosomes do not always preserve the telomeric sequences, and when retained these non-functional repeats could undergo a progressive degeneration or reduction [46], that could impede their detection by FISH.

### Sex chromosomes

The comparative analysis between the karyotypes of both sexes of *P. elegans* in addition to their meiotic behaviour showed a heteromorphism between males and females, which is compatible with the presence of an X_1_X_1_X_2_X_2_/X_1_X_2_Y sex chromosome system, in which the Y chromosome would correspond to the large metacentric chromosome exclusive to males, and the X_1_ and X_2_ chromosomes would correspond to two of the largest acrocentric chromosomes of the complement. According to this system females of *P. elegans* have 2n = 90 (86 + X_1_X_1_X_2_X_2_) whereas males have 2n = 89 (86 + X_1_X_2_Y).

Interestingly, the C-banding technique revealed a lack of constitutive heterochromatin in the sex chromosomes, not even in the Y chromosome which also turned out to be remarkably large.

Typically, during the evolution of sex chromosomes from autosomes, the reduction of recombination between the sex-determining regions is the first step to produce simple sex chromosome systems (XY or ZW). Then, the differential accumulation of repetitive sequences and deleterious mutations favour the heteromorphism between the X and Y (or Z and W), either in size, morphology or through banding techniques [47], and the recombination is kept in the pseudoautosomal regions of the sex chromosomes. In regard to the multiple sex chromosome systems, the initial stage of differentiation seems to be associated with chromosomal rearrangements between the chromosomes bearing sex-determining genes and an autosome (e.g. [48–50]). Consequently, due to rearrangements, even newly evolved sex chromosomes can be heteromorphic [51] and not necessarily involve heterochromatin increase [49]. These considerations may explain the existence of meiotic recombination between the *P. elegans* X and Y chromosomes, the lack of heterochromatin in them and the size of the euchromatic Y chromosome; indicating the possibility that the multiple sex chromosome system in this prawn species is a result of recent evolution. In light of this possibility, and bearing in mind male and female karyotypes and their meiotic behaviour, the initial step of sex chromosome differentiation in this species could be a centric fusion between two nonhomologous acrocentric chromosomes, forming the large metacentric neo-Y and leading to two acrocentric chromosomes without homologous in males (neo-X_1_ and X_2_ chromosomes). Accordingly, during meiosis, the recently formed neo-Y would pair with the neo- X_1_ at one end and with the neo-X_2_ at the other end, which would lead to the formation of a trivalent such as we observed.

In neither this nor our previous report [5], did we identify sex chromosomes in *P. serratus*. Also, we did not find differences in the constitutive heterochromatin pattern between sexes. Even so, the results demonstrated that the sex chromosome systems of both congeneric species are different since mitotic and meiotic metaphases displayed the same chromosome number in both *P. serratus* males and females, making a multiple sex determination system impossible in that species. In this regard, future studies involving comparative genomic hybridization would be helpful in investigating the putative absence of heteromorphic sex chromosomes in detail in the aforementioned species.

A review of the literature suggests that the multiple sex chromosome system X_1_X_1_X_2_X_2_/X_1_X_2_Y found in *P. elegans* may be unprecedented among decapods with the exception of *Cervimunida princeps* [52]. However, without additional studies using current techniques, the *C. princeps* sex determination system formulated in 1959 is questionable considering that it was based on male chromosome number (2n = 109) and the presence of three univalents at meiotic metaphase I, observations that could correspond for instance to an XX/XY_1_Y_2_ system.

The present data show the first karyotype with distinguishable heteromorphic sex chromosomes within the family Palaemonidae, where a ZZ/ZW sex chromosome system had been suggested for *Macrobrachium rosenbergii*, in which it is believed that the female is the heterogametic sex on the basis of molecular studies [53]. In fact, the ZZ/ZW sex-determining mechanism was never determined cytogenetically in any member of Decapoda although its existence has also been inferred in the crayfish species *Cherax quadricarinatus* (infraorder Astacidea) [54] and some penaeid shrimps (for a review, see [55, 56]). In contrast, male crabs (infraorder Brachiura) are reported to be the heterogametic sex based on their karyotype, with an XX/XY sex chromosome system and even an XX/XO system being observed (see the reviews [6, 57]). Notwithstanding, due to the inherent limitations of the techniques used at the time, we should be cautious as to the reliability of these studies. Recently, the ZZ/ZW sex determination system was proposed for the Chinese mitten crab *Eriocheir sinensis* (infraorder Brachiura) based on QTL mapping and confirmed by triploid induction experiments [58].

Our results on *Palaemon* sex determination systems and our bibliographic review reveal a large variability within Decapoda. They also show the difficulty of identifying sex chromosomes in this order using cytogenetic methods. The absence of heterochromatic blocks in the sex chromosomes in *P. elegans* could be a widespread characteristic in decapods. Besides, the high chromosome number and their small and homogenous size complicate the identification of sex chromosome pairs, especially if the meiotic stage, where the homologous are connected and the chromatin more condensed, is not analyzed.

## Conclusions

This and our previous study [5] show that the congeners *P. serratus* and *P. elegans* present a high degree of diversity in their chromosome number, karyotype and sex determination system, ranging from the putative absence of heteromorphic sex chromosomes to the multiple chromosome system (X_1_X_1_X_2_X_2_/X_1_X_2_Y). Such variability, even between species so closely related, makes this genus a promising model among Decapoda to investigate not only the karyotype evolution but also the patterns of sex chromosome differentiation.

In this perspective, future comparative cytogenetic analyses comprising other *Palaemon* species are needed to clarify the hypothesis developed in this work where fusions events would constitute the main mechanism of karyotype evolution in the genus. Likewise, the sex determination system in *P. serratus* and the existence of additional sex chromosome systems in the genus that shed light on the genus sex chromosome evolution are interesting aspects to be elucidated in further studies.
